# Molecular characterization of Golgi apparatus-related genes indicates prognosis and immune infiltration in osteosarcoma

**DOI:** 10.18632/aging.205645

**Published:** 2024-03-07

**Authors:** Jian Zhang, Jiahao Liu, Rui Ding, Xinxin Miao, Jianjian Deng, Xiaokun Zhao, Tianlong Wu, Xigao Cheng

**Affiliations:** 1 Department of Orthopedics, The Second Affiliated Hospital of Nanchang University, Nanchang 330006, Jiangxi, China; 2 Institute of Orthopedics of Jiangxi Province, Nanchang 330006, Jiangxi, China; 3 Institute of Minimally Invasive Orthopedics, Nanchang University, Nanchang 330006, Jiangxi, China

**Keywords:** osteosarcoma, Golgi apparatus, biomarkers, prognosis, immune infiltration

## Abstract

Background: The Golgi apparatus (GA) is crucial for protein synthesis and modification, and regulates various cellular processes. Dysregulation of GA can lead to pathological conditions like neoplastic growth. GA-related genes (GARGs) mutations are commonly found in cancer, contributing to tumor metastasis. However, the expression and prognostic significance of GARGs in osteosarcoma are yet to be understood.Methods: Gene expression and clinical data of osteosarcoma patients were obtained from the TARGET and GEO databases. A consensus clustering analysis identified distinct molecular subtypes based on GARGs. Discrepancies in biological processes and immunological features among the subtypes were explored using GSVA, ssGSEA, and Metascape analysis. A GARGs signature was constructed using Cox regression. The prognostic value of the GARGs signature in osteosarcoma was evaluated using Kaplan-Meier curves and a nomogram.

Results: Two GARG subtypes were identified, with Cluster A showing better prognosis, immunogenicity, and immune cell infiltration than Cluster B. A novel risk model of 3 GARGs was established using the TARGET dataset and validated with independent datasets. High-risk patients had poorer overall survival, and the GARGs signature independently predicted osteosarcoma prognosis. Combining risk scores and clinical characteristics in a nomogram improved prediction performance. Additionally, we discovered Stanniocalcin-2 (STC2) as a significant prognostic gene highly expressed in osteosarcoma and potential disease biomarker.

Conclusions: Our study revealed that patients with osteosarcoma can be divided into two GARGs subgroups. Furthermore, we have developed a GARGs prognostic signature that can accurately forecast the prognosis of osteosarcoma patients.

## INTRODUCTION

Osteosarcoma, a malignant tumor originating from bone tissue, primarily affects adolescents [1]. Patients’ survival rates have increased with current treatment choices, which include surgery, chemotherapy, and radiation therapy [2, 3]. Nevertheless, osteosarcoma patients develop metastases between 10 and 25% of the time they are diagnosed, and up to 90% of these metastases occur in the lungs [4, 5]. Despite the availability of various drugs, the prognosis for patients with advanced, metastatic, recurrent, or resistant osteosarcoma remains poor [6]. Given the limitations of conventional treatment approaches, there is an urgent need to develop innovative therapeutic strategies to increase the overall survival of osteosarcoma patients.

The Golgi apparatus (GA) is a critical organelle within the cell that is in charge of the modification, packaging, and transport of proteins from the endoplasmic reticulum to other cellular components [7]. In addition to its well-known roles, recent studies have discovered that GA regulates multiple cancer-related processes, including innate immune responses, angiogenesis, and cell proliferation [8–10]. Furthermore, GA-related genes (GARGs) have been discovered to be frequently altered in cancers, and such alterations likely to enhance tumor spread and result in a worse prognosis [11–14]. Improving our understanding of the GARGs pattern, and even the probable biochemical processes and mechanisms of osteosarcoma, is critical for improving therapeutic success.

In this study, transcription data and clinical details from 85 osteosarcoma patients were combined. Two GARGs clusters were comprehensively evaluated, and the relationship between different clusters and immune characteristics was systematically analyzed. Furthermore, an GARGs signature was constructed to determine the clinical prognosis of osteosarcoma, and validation was conducted in multiple independent datasets. Finally, we found Stanniocalcin-2 (STC2), a crucial prognostic gene that has significant expression in osteosarcoma and could serve as a promising biomarker for the disease.

## MATERIALS AND METHODS

### Data source

85 osteosarcoma patients’ RNA-seq data and clinical details were acquired from the TARGET database for consensus clustering and utilized as a training group for follow-up analysis. As verification cohorts, the GSE21257 and TCGA-SARC cohorts were employed, which included transcription data and linked clinical characteristics from 53 osteosarcoma patients and 262 sarcoma patients, respectively. The GSE225588 dataset (n=12) and GSE99671 dataset (n=36) were analyzed for differential gene expression. The 1643 GARGs were collected from the MSigDB gene collection “GOCC_GOLGI_APPARATUS”.

### Consensus clustering analysis

The prognostic GARGs were identified by univariate Cox regression analysis, and genes satisfying P<0.05 were screened. The “ConsensusClusterPlus” R software was then employed to classify GARGs molecular subtypes depending on the expression of these prognosis-related GARGs.

### Single-sample gene set enrichment analysis (ssGSEA) and gene set variation analysis (GSVA)

A ssGSEA method was used to calculate the levels of immune cell infiltration in each osteosarcoma sample [15]. Based on the “GSVA” algorithm, The gene collection “c2.cp.kegg.v7.4.symbols” gathered through MSigDB was applied to show alterations in signaling pathways among two subtypes.

### Differentially expressed genes (DEGs) and Metascape enrichment analysis

The “limma” R package was utilized to conduct an analysis of the DEGs between the two GARGs subtypes, with FDR threshold of less than 0.05 and a logFC greater than 1. The gene enrichment analysis was conducted utilizing the Metascape webtool, a widely recognized bioinformatics platform (http://metascape.org/). Subsequently, the above DEGs were selected for a consensus clustering analysis.

### The GARGs prognostic signature

The prognostic GARGs were subjected to analysis through the application of LASSO regression, as well as multivariate Cox regression analysis. Following that, three genes were identified and utilized to generate a risk score. The following formula was used to get the risk score:


$$risk\;score=\sum_{i=1}^{n}\left(Coef_{i}*x_{i}\right)$$

Here, *Coef_i_* denotes the gene coefficients, while *x_i_* indicates the corresponding gene expression levels.

The patient group was separated into groups with high and low risk based on the optimal cut-off value. The “survival” R package was applied to analyze the association between survival and the GARGs signature. The prognostic value was determined using ROC curves created with the “timeROC” R package.

### Independent analysis and nomogram

Univariate and multivariate Cox regression analyses were applied to investigate the independent prognostic factors, comprising age, gender, and metastasis. The “rms” package was utilized to construct a nomogram and generate calibration curves to evaluate the consistency between anticipated and observed outcomes.

### Estimation of tumor immune microenvironment

The CIBERSORT method was employed to measure the abundance of 22 distinct immune cell subtypes, while the ESTIMATE method was applied to quantify the tumor microenvironment scores of osteosarcoma patients, encompassing ESTIMATE, immune, and stromal scores. Then, we compared these scores between the two different risk groups.

### Pan-cancer analysis of STC2

The expression levels and prognostic role of STC2 were visualized across several common types of cancer through the GEPIA webtool (http://gepia.cancer-pku.cn/).

### Cell source and culture

The osteosarcoma cell lines (143B, MG63, HOS) and the human fetal osteoblast cell line (hFOB 1.19) were procured from the Cell Bank of the Chinese Academy of Sciences (Shanghai, China). DMEM supplemented with 10% fetal bovine serum (Gibco, USA) and 1% penicillin/streptomycin (Solarbio, China) was used to cultivate the cells. The osteosarcoma cells were maintained at 37° C and 5% CO2, while the hFOB 1.19 were cultivated at 34° C with a 5% CO2 concentration.

### RNA isolation and real-time fluorescent qPCR

TRIzol™ Reagent (Thermo Fisher Scientific, USA) was used to extract total RNA from cells, and the PrimeScriptTM RT Reagent Kit (Perfect Real Time) (Takara, Japan) was applied for synthesis of cDNA. For real-time PCR analysis, GAPDH was employed as a control. Gene primers are listed in Supplementary Table 1.

### Clinical specimens

Three osteosarcoma samples were obtained, as well as three matched adjacent normal tissues. The samples were collected from individuals with osteosarcoma who had surgery at The Second Affiliated Hospital of Nanchang University. The study was ethically approved by the research ethics committee of the aforementioned hospital, and all patients provided informed consent.

### Immunohistochemical staining

Immunohistochemistry was used to measure the levels of STC2 according to standard procedures. Tissue sections were stained with rabbit anti-STC2 antibody (10314-1-AP, 1:200, Proteintech, China) as the primary antibody, followed by HRP-conjugated goat anti-rabbit IgG antibody. The images were captured using a microscope (COIC, XSP-C204, China).

### Statistical analysis

The statistical analyses were performed using R 4.0.4 and SPSS Statistics 25. To compare the two groups, Student’s t-test was used, and statistical significance was assessed at a p-value of 0.05.

### Data availability

The datasets supporting the conclusions of this article are available in the TARGET-OS (https://ocg.cancer.gov/programs/target), the Gene Expression Omnibus (https://www.ncbi.nlm.nih.gov/geo/), the TCGA database (https://portal.gdc.cancer.gov/), Molecular Signatures Database (MSigDB, http://www.gsea-msigdb.org/gsea/msigdb/).

## RESULTS

### Identification of GARGs molecular subtypes

**Figure 1:**
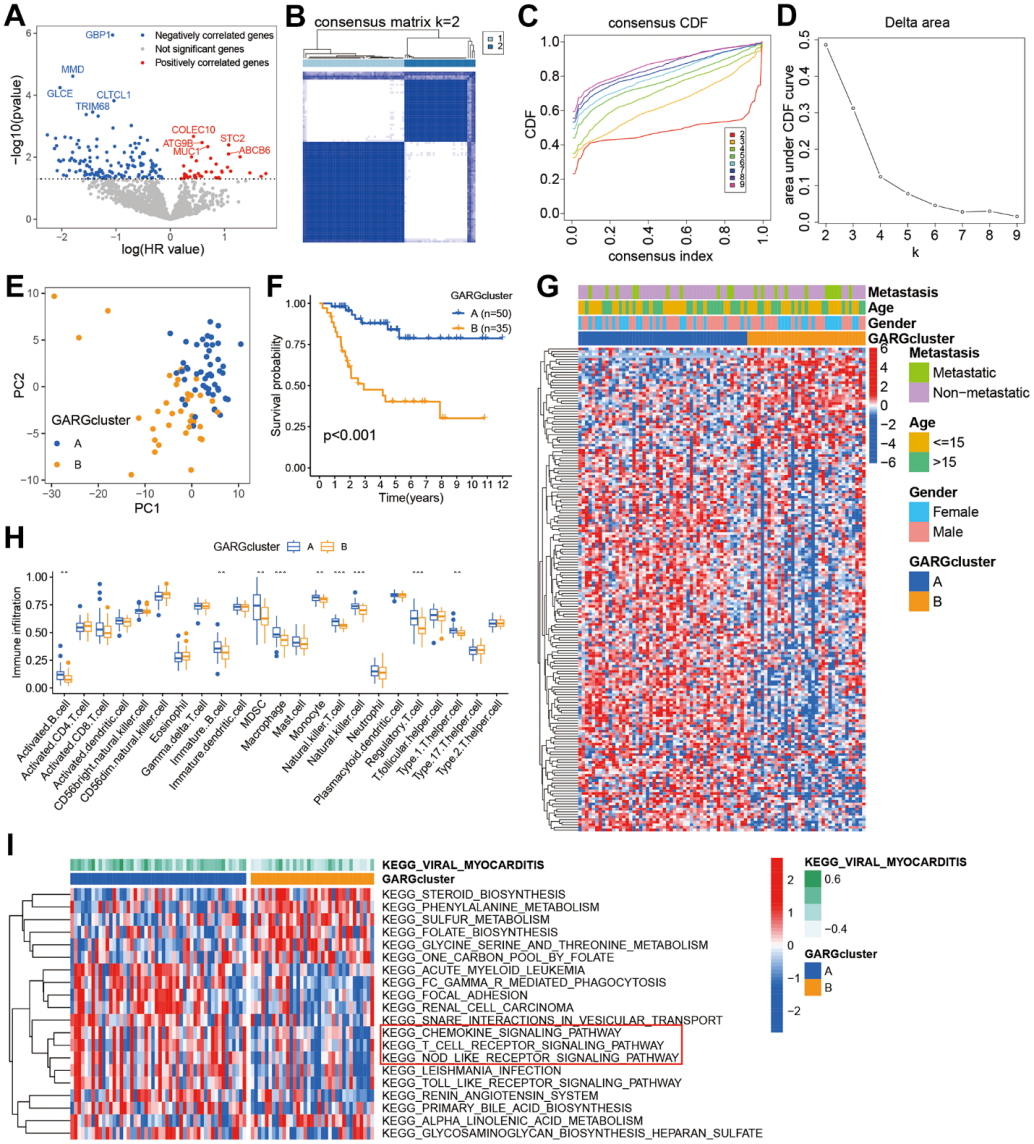
**Prognostic gene screening and consensus clustering.** (**A**) Volcano plot displaying univariate Cox regression results of GARGs. (**B**) The consensus matrix of 85 samples when *k* = 2. (**C**, **D**) The CDF curve for *k* = 2-9. (**E**) PCA plot of the two subtypes. (**F**) Kaplan-Meier survival analysis of the two subtypes. (**G**) Comparison of GARGs expression and clinical characteristics between the two subtypes. (**H**) The immune cell infiltration between the two subtypes was analyzed by the ssGSEA. *P<0.05, **P<0.01 and ***P<0.001. (**I**) GSVA was performed to analyze the differences between the two subtypes.

The GARGs expression matrix was extracted from the TARGET datasets, followed by the execution of univariate Cox analysis to explore the genes linked to prognosis. The results revealed that 186 GARGs were significantly (p <0.05) associated with prognosis (Figure 1A and Supplementary Table 2). Consensus clustering analysis identified two subtypes, 50 of the patients were assigned to cluster A, and 35 patients were assigned to cluster B (Figure 1B–1D). Principal component analysis (PCA) showed a significantly different distribution between the two clusters (Figure 1E). Additionally, the findings of the survival study revealed a substantial difference in overall survival times between the two subtypes, with patients in cluster B having a much worse overall survival than those in cluster A (Figure 1F). The heatmap showed GARGs expression and clinical features in osteosarcoma, with Cluster A exhibiting considerably greater expression of most GARGs (Figure 1G). According to the ssGSEA results, we found disparities in the infiltration of most immune cells between the two subtypes, such as activated B cell, immature B cells, Macrophage, and so on, between the two subtypes (Figure 1H). We used the GSVA to further explore the biological behavior between the two subtypes. Cluster A was shown to be a highly enriched pathway linked with immunological function, such as chemokine signaling pathway, T cell receptor signaling pathway, and NOD-like receptor signaling pathway (Figure 1F). These findings suggest that unsupervised clustering based on GARGs distribution can classify osteosarcoma into two clusters with different clinical prognostic and tumor immunophenotypic characteristics.

### Comprehensive analysis of DEGs associated with GARGs subtypes

**Figure 2:**
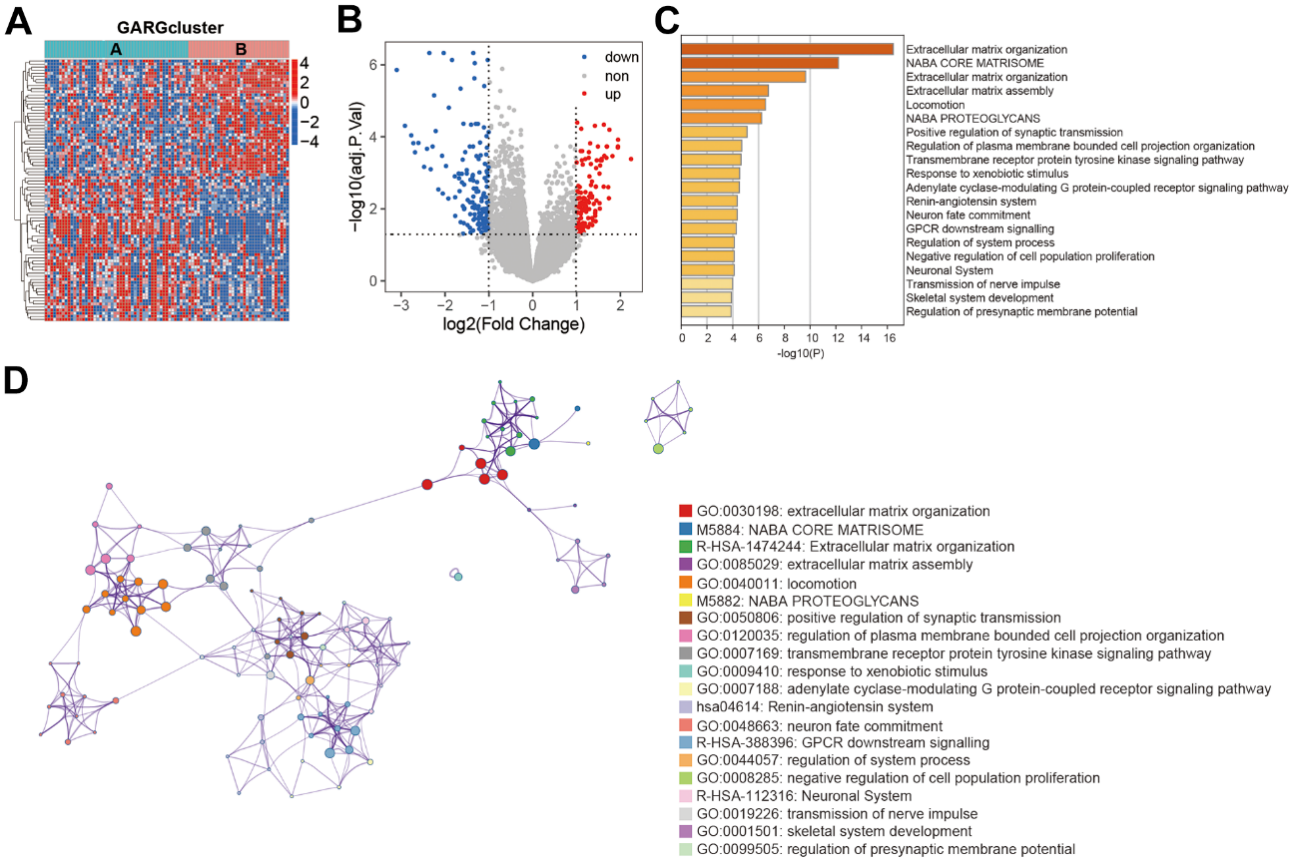
**Functional enrichment analysis between the two subtypes.** (**A**) Heatmap of DEGs between the two subtypes. (**B**) The volcano plot of DEGs. (**C**) Biological process and pathway enrichments ordered by statistical significance. (**D**) The network showed the interactions among the enriched terms.

267 DEGs were discovered between the two GARGs subtypes to better elucidate the functional role of the GARGs patterns described above. The resulting heatmap and volcano plots of these DEGs are presented in Figure 2A, 2B. Subsequent enrichment analysis of these DEGs, utilizing the Metascape webtool, revealed that they were predominantly enriched in the extracellular matrix organization, positive regulation of synaptic transmission, Naba core matrisome (Figure 2C, 2D).

**Figure 3:**
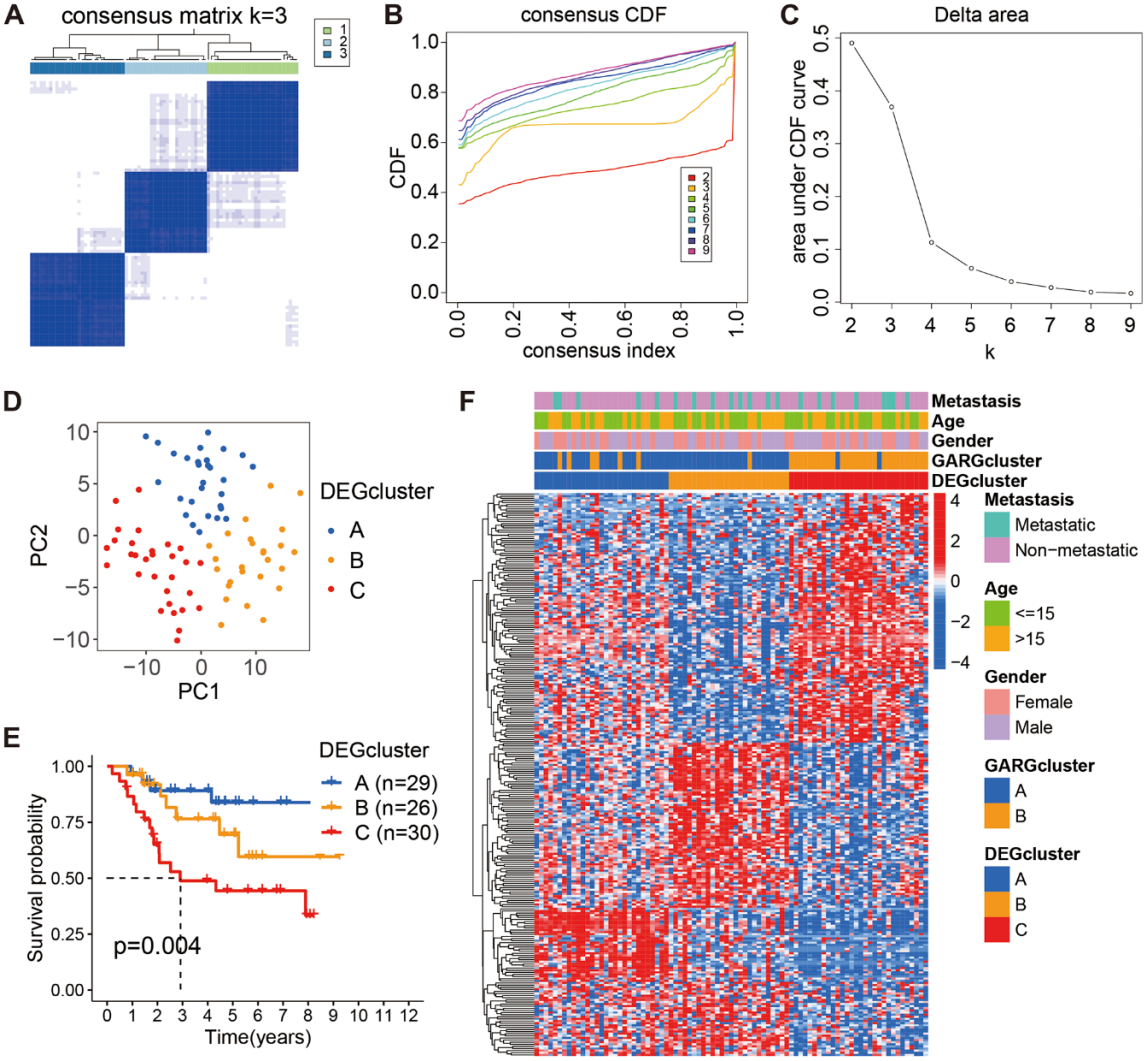
**Consensus cluster analysis based on the DEGs.** (**A**) The consensus matrix when *k* = 3. (**B**, **C**) The CDF curve for *k* = 2-9. (**D**) PCA plot of the three subtypes. (**E**) Kaplan-Meier survival analysis of the three subtypes. (**F**) Comparison of DEGs expression and clinical characteristics between the three subtypes.

Furthermore, based on these 267 DEGs, we employed a consistent clustering approach to divide osteosarcoma patients into three gene clusters (Figure 3A–3C). PCA results showed the existence of different distributions of the three gene clusters (Figure 3D). According to the K-M survival results, gene cluster A had much greater long-term survival than gene cluster C (Figure 3E). Moreover, the heatmap demonstrated the expression of GARGs as well as clinical features in osteosarcoma (Figure 3F). As a result, these findings verified the reliability of the GARGs cluster and showed a critical function for GARGs in osteosarcoma.

### Construction and evaluation of the GARGs signature

**Figure 4:**
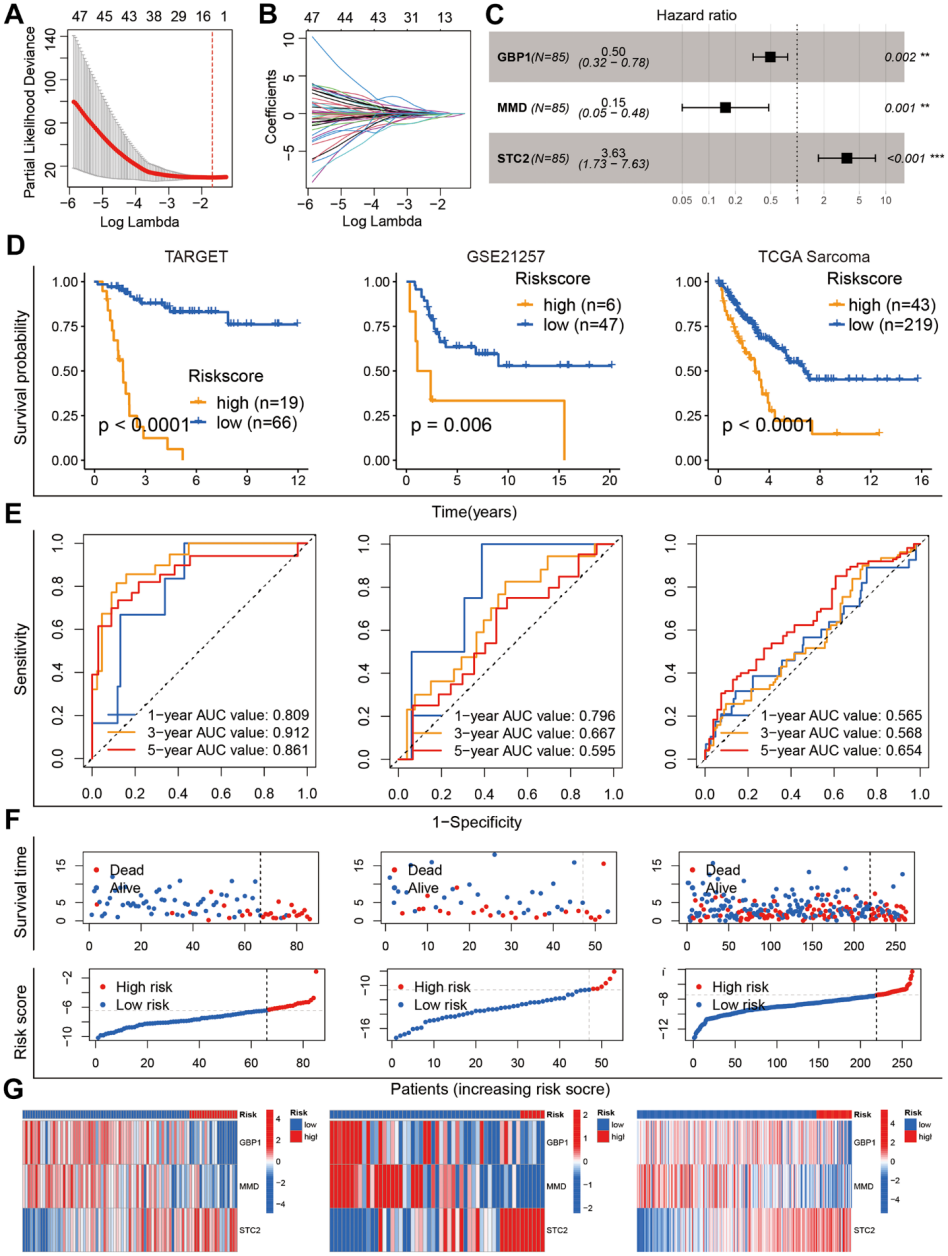
**Construction and validation of the GARGs risk model.** (**A**, **B**) LASSO regression analysis of 186 prognostic GARGs. (**C**) Multivariate Cox regression analysis. (**D**) Kaplan-Meier curves in the TARGET, GSE21257 and TCGA-SARC cohorts. (**E**) The AUC for the prediction of 1, 3, 5 years survival rate. (**F**) Distribution of survival status and risk scores. (**G**) Heatmap of the three model genes between the high- and low-risk groups.

The 186 prognostic GARGs were subjected to LASSO Cox regression analysis (Figure 4A, 4B) followed by the construction of the GARGs signature through multivariate Cox regression analysis, and three genes and their risk coefficients were identified: GBP1, MMD, and STC2 (Figure 4C). According to the survival analysis, higher risk scores in the three cohorts strongly predicted poorer outcomes (Figure 4D). The ROC curves showed that GARGs signature were robust predictors of overall survival (Figure 4E). In addition, the distribution of risk scores, survival status distribution and gene expression profiles were consistent across the three patient cohorts (Figure 4F, 4G). Referring to the findings, our GARGs score can be utilized as an evaluation index for patient prognosis.

### Independent prognostic analysis and construction of nomograms

**Figure 5:**
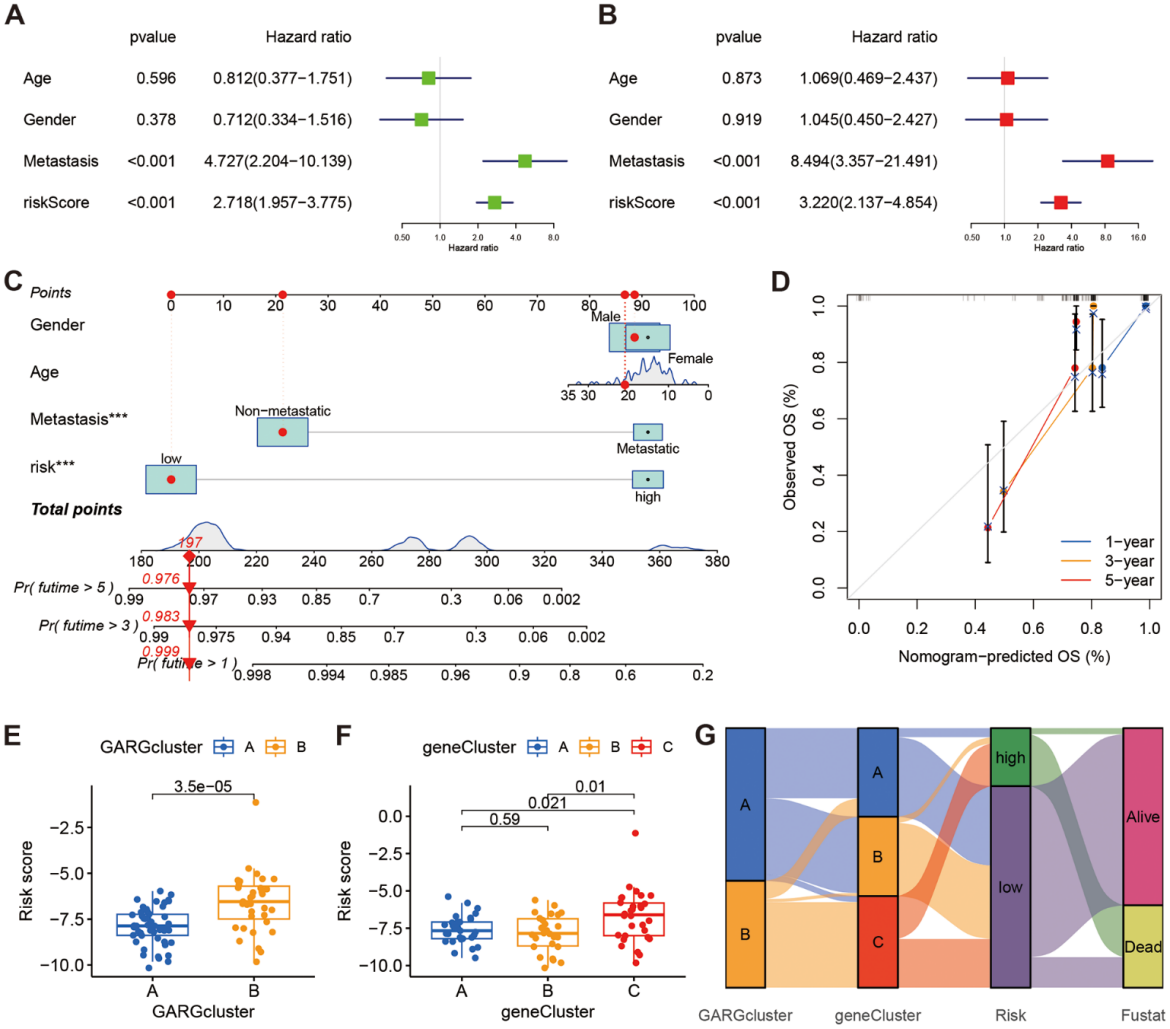
**The relationships between clinical characteristics and the GARGs signature.** (**A**, **B**) Univariate and multivariate Cox regression analysis for independent prognostic analysis of risk model. (**C**) Nomogram based on gender, age, metastasis and risk in the TARGET cohort. (**D**) The nomogram calibration curves for predicting 1-, 3-, and 5-year survival. (**E**) The relationship between risk score and the two GARGs subtypes. (**F**) The relationship between the risk score and the three DEGs subtypes. (**G**) Sankey plot of GARGs subtype distribution in groups with different risk scores and survival status.

We found a significant association between risk score and overall survival, as evidenced by both univariate and multivariate Cox analyses (Figure 5A, 5B). The risk score was then combined with additional clinicopathological risk variables to form a nomogram, which revealed it to be the most significant component among the other clinical factors (Figure 5C). Notably, the calibration curves demonstrated a high degree of concordance between anticipated and observed survival rates at 1, 3, and 5 years (Figure 5D). Also, we examined the relationship between risk scores and subtypes, and box plots showed high risk score in both GARGs subtypes B and DEGs subtypes C (Figure 5E, 5F). Additionally, a Sankey diagram was employed to illustrate the distribution of the population across two GARGs subtypes, three DEGs subtypes, and two GARGs risk score groups (Figure 5G).

### Immunity statue in distinct GARGs risk score groups

**Figure 6:**
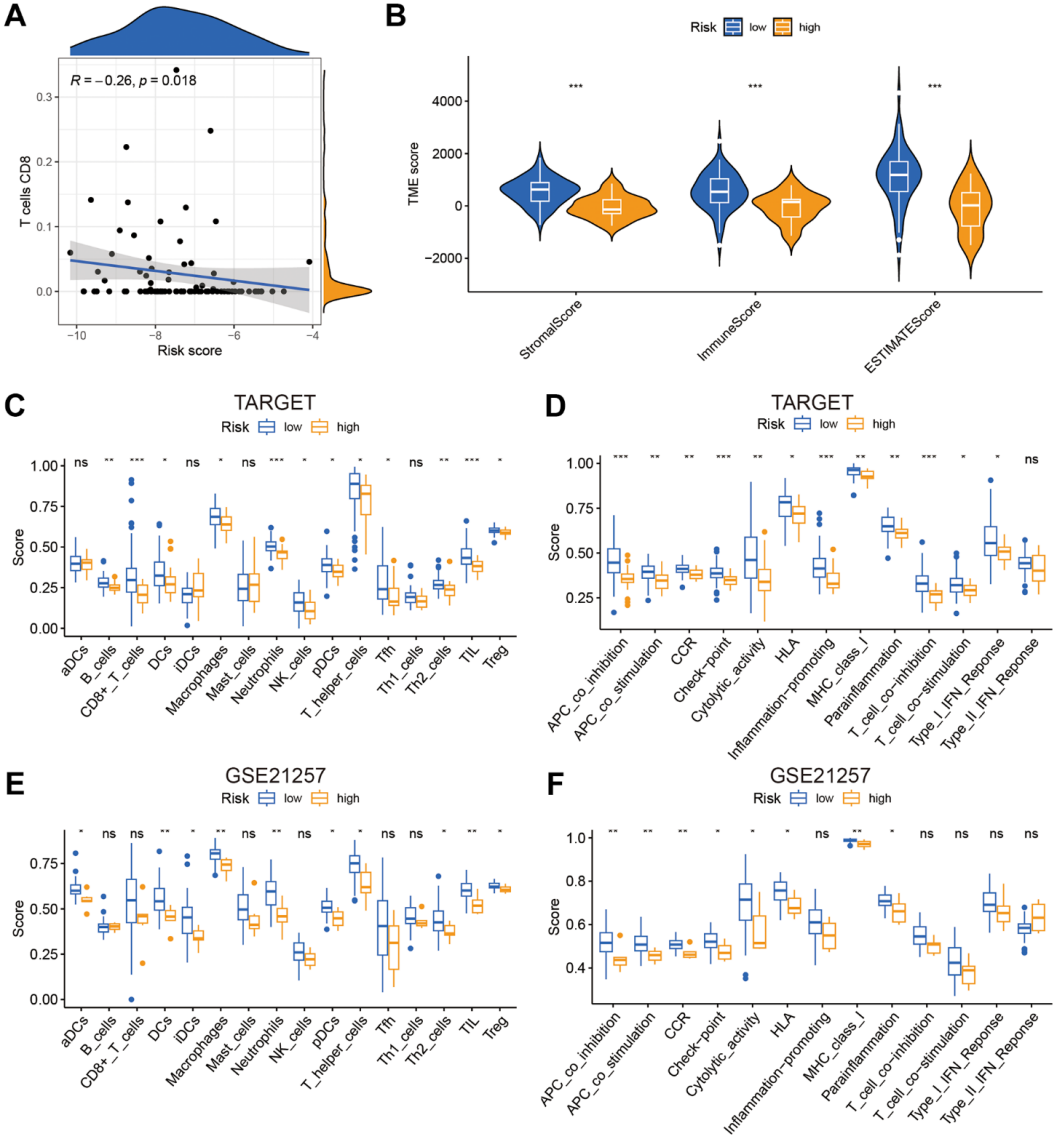
**The risk score was related to immune infiltration.** (**A**) The association between risk score and immune cell infiltration. (**B**) Risk score was significantly correlated with stromal scores, immune scores, and ESTIMATE scores. (**C**–**F**) Relationship between risk score and immune cell infiltration and related functions via ssGSEA analysis. *P<0.05, **P<0.01 and ***P<0.001.

T lymphocytes CD8 were found to be negatively connected with risk score using Pearson correlation analysis (Figure 6A). Then, for each sample, we used the R package “estimate” to compute the stromal score, immune score, and ESTIMATE score. Risk scores were significantly inversely associated to stromal, immune, and ESTIMATE scores (Figure 6B). According to the ssGSEA results displayed in Figure 6C–6F, the infiltration of immune cells and associated functions decreased in the high scoring group. According to the findings, patients with a low-risk score had a better prognosis and higher immune cell infiltration.

### STC2 is highly expressed in osteosarcoma

**Figure 7:**
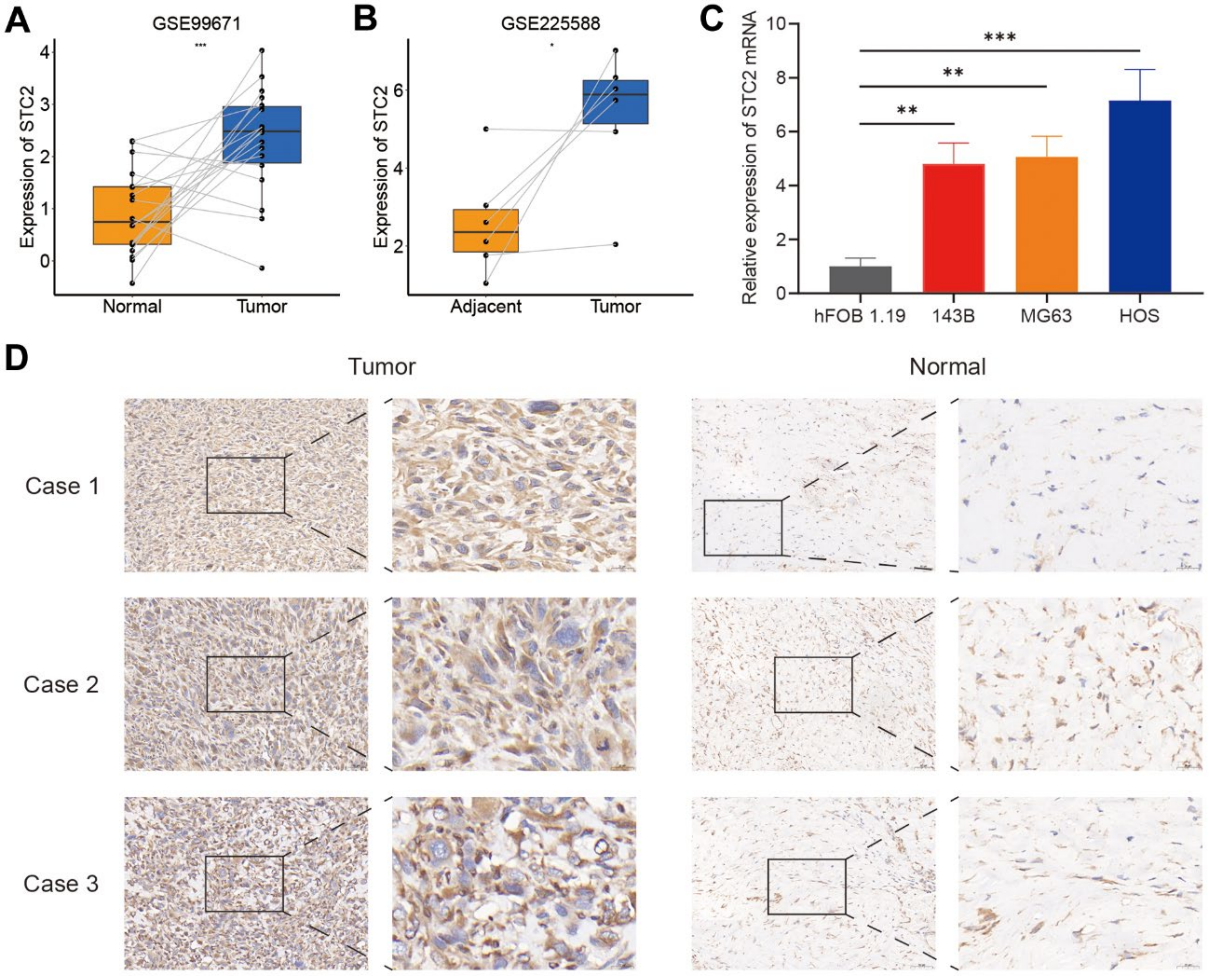
**The expression levels of STC2.** (**A**, **B**) The STC2 expression level in osteosarcoma and non-tumoral paired samples, based on the GSE99671 and GSE225588 cohort. (**C**) The qRT-PCR result of STC2 in hFOB 1.19, 143B, MG63, HOS cell lines. (**D**) The expressions of STC2 in tumor and adjacent normal tissues. *P<0.05, **P<0.01 and ***P<0.001.

Since STC2 was the only upregulated gene in the high-risk group, it was chosen as a focus for further investigation. The STC2 expression in osteosarcoma specimens and control tissue was compared using two separate datasets from the GEO database (GSE225588 and GSE99671). STC2 expression was shown to be significantly upregulated in osteosarcoma specimens (Figure 7A, 7B). The qRT-PCR results revealed that STC2 expression levels in osteosarcoma cells were much higher than in osteoblasts (Figure 7C). By immunohistochemistry staining, we identified a difference in STC2 expression in three pairs of osteosarcoma tissues and adjacent control tissues, and the results were comparable with the mRNA data (Figure 7D).

### The pan-cancer analysis of STC2

**Figure 8:**
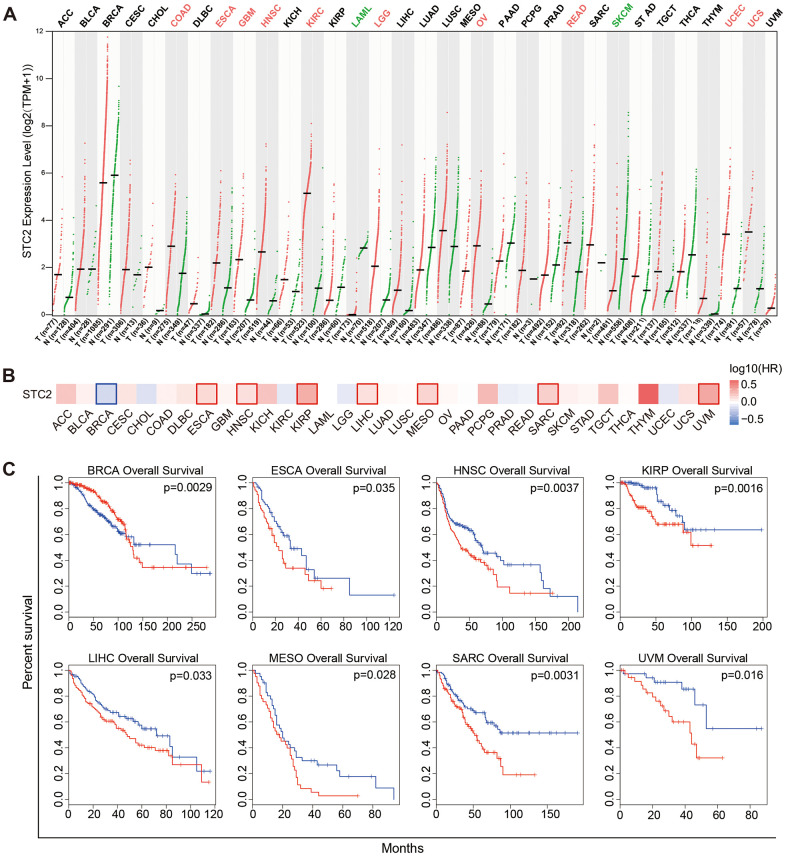
**Expression and significance of STC2 in pan-cancer.** (**A**) Pan-cancer analysis of STC2 expression based on the GEPIA2. (**B**) Survival map of STC2 in pan-cancer. (**C**) Kaplan-Meier survival curves for overall survival rate over TCGA cancer types.

STC2 expression trends in pan-cancer has been explored further. The results revealed high expression of STC2 in the majority of tumors, such as COAD, ESCA, GBM, HNSC, KIRC, LGG, OV, READ, UCEC, UCS and low expression in LAML, SKCM, indicating the significance of STC2 as a crucial biomarker for pan-cancer (Figure 8A). Increased STC2 expression is linked to poor survival in a number of malignancies, including ESCA, HNSC, KIRP, LIHC, MESO, SARC, and UVM, according to a survival analysis of tumors (Figure 8B, 8C).

## DISCUSSION

Osteosarcoma is a frequently aggressive neoplasm affecting children and adolescents, with a low rate of survival [16]. The GA has a crucial role in tumor microenvironment, malignant progression, and pharmacotherapy [9, 17, 18]. GARGs mutations are widespread in most tumors, and their presence can promote tumor metastasis [19–21]. Consequently, the current investigation was formulated to comprehensively and systematically assess the prognostic and therapeutic implications of GARGs in osteosarcoma.

The present study involved the initial classification of osteosarcoma into two subgroups, predicated upon the GARGs related to prognosis. The results indicated that Cluster A exhibited a notably superior disease prognosis in comparison to Cluster B. Recent research has indicated that the GA serves not only as a mediator of protein and lipid processing and transportation, but also as a signaling hub for innate immune signaling and subsequent effector responses [22–24]. Consequently, we conducted an investigation into the infiltration of immune cells across distinct subtypes. Our findings demonstrated a significant variation in the immunological microenvironment between the two subtypes, which may contribute to the observed disparity in survival rates among patients belonging to these subtypes.

Furthermore, the amalgamation of GARGs subtypes analysis with biomarker identification has the potential to open up new routes for future investigation, ultimately enhancing the prognostic outcomes of patients [25]. In this work, a model including three GARGs were constructed and verified in two independent cohorts. Through various ways, the three genes influence tumor aggressive behavior. GBP-1 is a constituent of gene signatures that exhibit a positive correlation with enhanced prognosis of breast cancer [26, 27]. Furthermore, GBP-1 impedes the growth of tumor cells by restraining angiogenesis [28, 29]. Additionally, it functions as an upstream regulator of various types of cell death in macrophages and stimulates the activation of microbe-specific downstream pathways [30]. MMD, or called PAQR11, is a member of progestin and AdipoQ receptor (PAQR) family [31]. PAQR11 activates Ras signalling by acting as a scaffold protein in the GA [32]. Recent study has linked higher PAQR11 levels to epithelial-to-mesenchymal transition (EMT) and poor survival in human cancers [33]. Additionally, PAQR11 has been identified as a crucial factor in tumor cell migration and metastasis in EMT-driven lung adenocarcinoma models [33]. STC2 is a secreted glycoprotein that exists in a range of tissues and has been linked to physiological functions [34, 35]. STC2 has been implicated in cancer formation in a number of recent studies [36–38]. Furthermore, STC2 was found to modulate osteosarcoma cell proliferation, apoptosis, and EMT [39].

While the study yielded promising results, it is important to note that there were several limitations. Although the results were validated in various cohorts, prospective studies are required to corroborate the predictive markers found. Additionally, mechanistic analysis is required to establish the associations between immune infiltration and GARGs phenotype. Therefore, further functional experimental research is warranted in the future.

## CONCLUSIONS

In summary, this study examined the expression of GARGs in osteosarcoma and identified distinct patterns and signatures that are linked to immune infiltration. The GARGs signature not only correlates with the prognosis of osteosarcoma patients, but also with pathway activities and immune cell infiltrations, thereby presenting a novel avenue for therapeutic intervention in the management of osteosarcoma.

## Supplementary Material

Supplementary Table 1

Supplementary Table 2
