# Supplementary material for: Molecular characterization of Golgi apparatus-related genes indicates prognosis and immune infiltration in osteosarcoma
Source: Aging (Albany NY). 2024 Mar 7;16(6):5249–63. doi: 10.18632/aging.205645 (PMC11006476; doi:10.18632/aging.205645)
Supplement: Supplementary Table 1 [file aging-16-205645-s001.pdf]

SUPPLEMENTARY TABLE

Supplementary Table 1. Primers used in this study.

Gene	Forward primer 5'-3'	Reverse primer 5'-3'
STC2	AGGAGGAAGAGGAGGAGGAGGAAG	CCGCTCGGCACACATGGTTC
